# MGF360-9L Is a Major Virulence Factor Associated with the African Swine Fever Virus by Antagonizing the JAK/STAT Signaling Pathway

**DOI:** 10.1128/mbio.02330-21

**Published:** 2022-01-25

**Authors:** Keshan Zhang, Bo Yang, Chaochao Shen, Ting Zhang, Yu Hao, Dajun Zhang, Huanan Liu, Xijuan Shi, Guoli Li, Jinke Yang, Dan Li, Zixiang Zhu, Hong Tian, Fan Yang, Yi Ru, Wei Jun Cao, Jianhong Guo, Jijun He, Haixue Zheng, Xiangtao Liu

**Affiliations:** a State Key Laboratory of Veterinary Etiological Biology, National Foot-and-Mouth Disease Reference Laboratory, Lanzhou Veterinary Research Institute, Chinese Academy of Agricultural Sciences, Lanzhou, China; ARS, USDA; Yonsei University

**Keywords:** ASFV, MGF360-9L, JAK/STAT signal pathway, STAT1/2, virulence factor, immune escape, in vivo, in vitro

## Abstract

African swine fever (ASF)—an aggressive infectious disease caused by the African swine fever virus (ASFV)—is significantly unfavorable for swine production. ASFV has a complex structure and encodes 150–167 proteins; however, the function of most of these proteins is unknown. This study identified ASFV MGF360-9L as a negative regulator of the interferon (IFN)-β signal. Further evidence showed that MGF360-9L interacts with signal transducer and activator of transcription (STAT) 1 and STAT2 and degrades STAT1 and STAT2 through apoptosis and ubiquitin–proteasome pathways, respectively. Subsequently, the activation of IFN-β signaling was inhibited. Naturally isolated or genetically manipulated live attenuated viruses are known to protect against the virulent parental ASFV strains. Therefore, through homologous recombination, we deleted *MGF360-9L* from the virulent ASFV CN/GS/2018 strain to construct a recombinant strain, ASFV-Δ360-9L. Compared with the parent ASFV CN/GS/2018 strain, the replication level of ASFV-Δ360-9L decreased in primary porcine alveolar macrophage cultures at 24 h postinfection, but the difference is unlikely to be biologically relevant. Notably, ASFV-Δ360-9L was partially attenuated in pigs. To our knowledge, this study is the first to uncover the function of MGF360-9L during ASFV infection. MGF360-9L inhibits IFN-β signaling through the targeted degradation of STAT1 and STAT2. Furthermore, MGF360-9L is a key virulence gene of ASFV. Our findings reveal a new mechanism by which ASFV inhibits host antiviral response; this might facilitate the development of live attenuated ASFV vaccines.

## INTRODUCTION

African swine fever (ASF) is an infectious disease caused by the African swine fever virus (ASFV), a large double-stranded DNA virus that replicates in the cytoplasm and jeopardizes the production of pigs. ASFV is the only member of the *Asfarviridae* family and the only DNA arbovirus (1, 2). It is associated with high morbidity and mortality in domestic pigs (3). The first outbreak of ASF was reported in China in 2018, which caused a substantial economic loss for the Chinese pig industry and seriously threatened ecological security (4, 5). To the best of our knowledge, no safe and effective commercial vaccine has been developed against ASFV yet. This virus primarily targets cells of the mononuclear phagocytic system. Its ability to infect macrophages appears to be a critical factor in the virulence of ASFV (6).

Previous studies have suggested that the ASFV Armenian/07 strain inhibits interferon (IFN)-β production through the cGAS–STING pathway (7). Type I interferons (IFNs) are the first line of defense against viral infection. First, the host’s pattern recognition receptors recognize the evolutionarily conserved pathogen-associated molecular patterns (8). Next, the host signaling pathways are activated, triggering the expression of type I IFNs. These IFNs act on their corresponding receptors as well as activate and phosphorylate Janus kinase (JAK) 1 and tyrosine kinase 2 (TYK2). Activated tyrosine kinases, in turn, phosphorylates signal transducer and activator of transcription (STAT) 1 and STAT2. The phosphorylated STAT1 and STAT2 then interact with IFN regulatory factor 9 (IRF9) to form IFN-stimulated gene factor (ISGF) 3. ISGF3 can enter the nucleus and further enhance the activity of IFN-stimulated response element (ISRE) promoter, thus promoting the expression of IFN-stimulated genes (ISGs); thus, IFN expression plays a role in host antiviral response (9). Over the years, viruses have devised various strategies to inhibit the expression of downstream ISGs through the inhibition of the JAK/STAT pathway (10). For example, rotavirus avoids the host immunity through the nonstructural protein 1 (NSP1)-mediated degradation of IRF9 and inhibition of IFN-mediated STAT1 phosphorylation (11, 12). Recently, it has been reported that ASFV induces the degradation of STAT1 and STAT2 to antagonize Type I IFNs signaling (13) but which viral proteins play a role in it is still unknown.

ASFV has been reported to encode and express various immune escape proteins to suppress the host immune response, thus creating a favorable condition for self-proliferation and diffusion (14, 15). The ASFV genome contains several unique multigene families (MGFs): MGF100, MGF110, MGF300, MGF360, and MGF530/505 (16). The MGF360 family is located in the highly variable region at the left and right end of the whole ASFV genome structure (17). Members of the MGF360 family have been implicated in virus virulence and have thus been targeted for the development of live attenuated ASFV vaccines (18, 19). ASFV *MGF360* and *MGF530/505* genes play an essential role in the host range of macrophages (20). Previous evidence has shown that *MGF360* and *MGF530* family participate in the pathogenicity of ASFV in pigs (21). Burrage et al. highlighted that the ASFV MGF360 family is an important determinant of the host range of ticks (22). The deletion of multiple genes in MGF360 and MGF530/505 families can increase the expression of ISGs and type I IFNs in infected macrophages (6, 23). Currently, the functions of most of the genes in the multigene families are unknown; thus, further research is necessary.

The present study identified MGF360-9L, a member of the MGF360 family, as an inhibitor of the JAK/STAT pathway. ASFV MGF360-9L inhibits the IFN-β-induced ISG transcription by interacting with and degrading STAT1 and STAT2 proteins. We found that the deletion of *MGF360-9L* from the parent ASFV CN/GS/2018 strain (recombinant strain: ASFV-Δ360-9L) led to attenuated of the virus upon challenge with pigs. Thus, this study clarified the function of *MGF360-9L* and identified a new mechanism of ASFV involved in the evasion of host innate immunity.

## RESULTS

### ASFV inhibits IFN-β-induced ISGs transcription.

**FIG 1:**
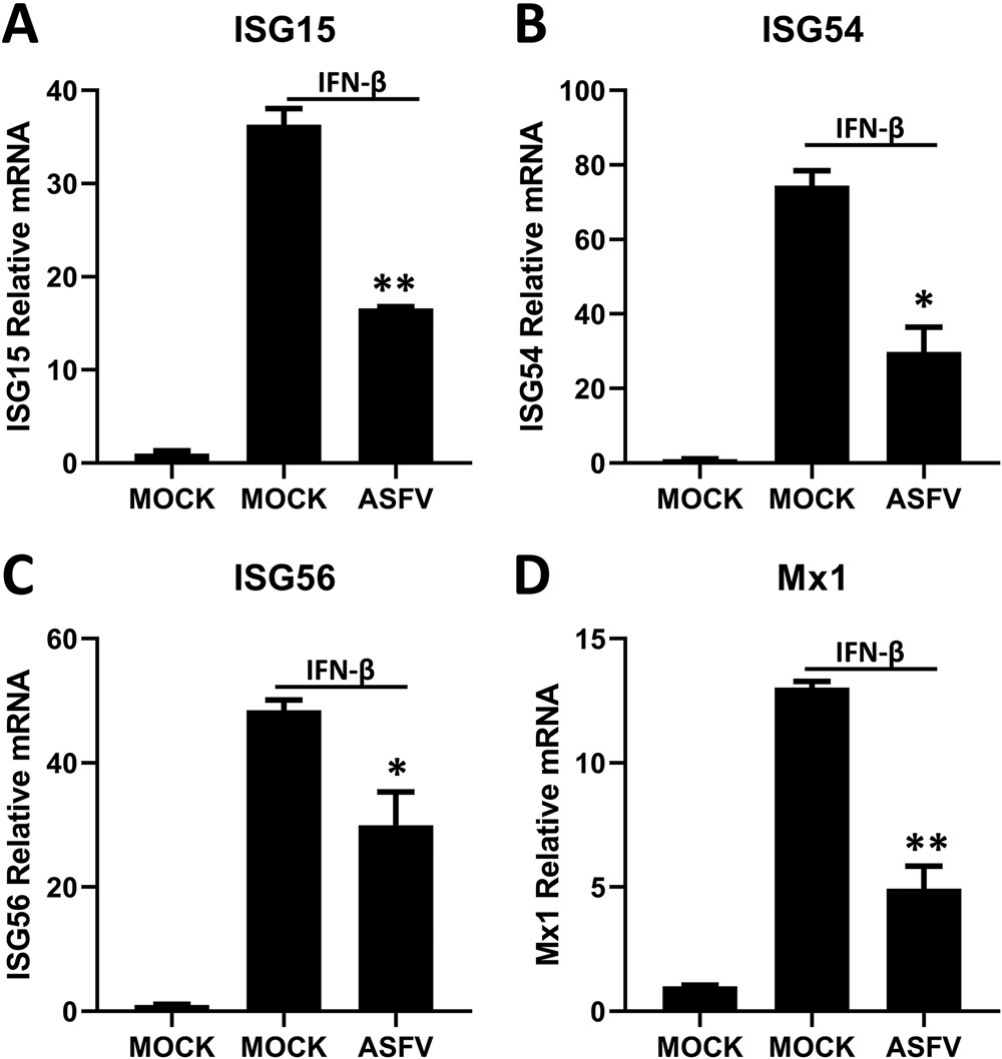
ASFV-WT inhibits IFN-β signaling. PAMs were cultured in a 12-well plate and infected with ASFV (MOI = 0.1) for 8 h. IFN-β (1,000 U/ml) was added to treat the cells for 8 h before analyzing the mRNA of ISG15 (A), ISG54 (B), ISG56 (C), and Mx1 (D) using RT-qPCR. Data are presented as the means ± SDs from three independent experiments.

IFN-β induces a strong antiviral response by inducing the expression of ISGs, thus controlling viral infection (9). To explore the mechanism that ASFV employs to inhibit IFN-β signaling, porcine alveolar macrophages (PAMs) were infected with ASFV and then treated with IFN-β. Afterward, the mRNA levels of ISG15, ISG54, ISG56, and IFN-induced GTP-binding protein Mx1 (*Mx1*) triggered by IFN-β were analyzed using quantitative reverse transcription-PCR (RT-qPCR). The results suggested that ASFV significantly inhibited IFN-β-induced ISG transcription at 8 h postinfection (hpi; Fig. 1). Thus, some proteins encoded by ASFV may inhibit the signal transduction of IFN-β.

### ASFV MGF360-9L inhibits IFN-β signaling.

**FIG 2:**
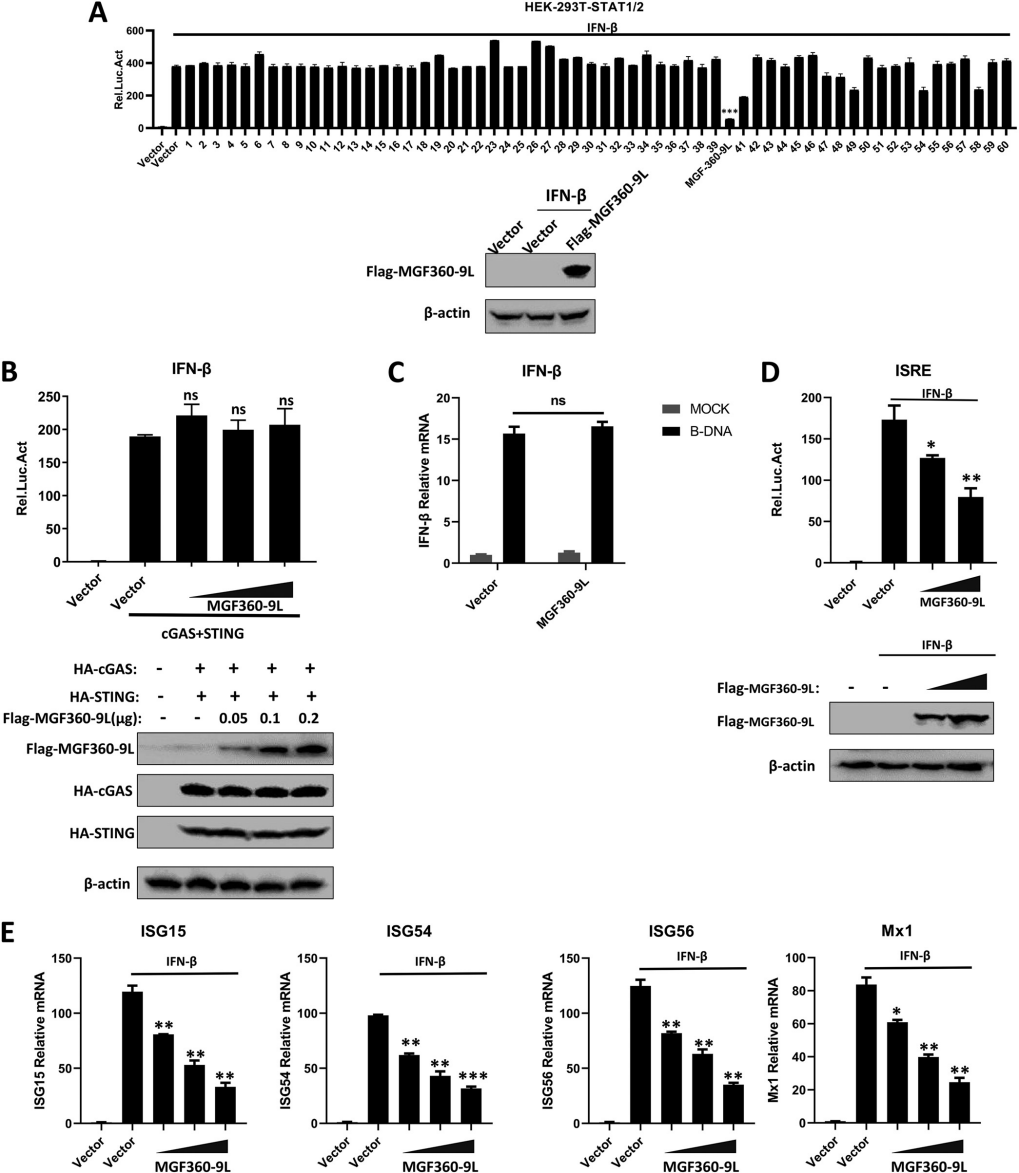
ASFV MGF360-9L inhibits IFN-β signaling. (A) HEK-293T cells cultured in 48-well plates were transfected with different ASFV protein expression plasmids (0.1 μg/well) along with STAT1/2-Luc plasmid (0.1 μg/well) and pRL-TK plasmid (0.005 μg/well). After 24 h, cells were treated with IFN-β (1,000 U/ml) for 8 h, and then a luciferase assay was performed. The expression of *MGF360-9L* was confirmed through Western blotting. (B) The HEK-293T cells cultured in 48-well plates were transfected with various concentrations of ASFV MGF360-9L expression plasmid (0, 0.05, 0.1, and 0.2 μg/well) along with HA-*cGAS* (0.1 μg/well), HA-*STING* (0.1 μg/well), *IFN-β-Luc plasmid* (0.05 μg/well), and pRL-TL (0.005 μg/well). After 24 h of transfection, the luciferase activity was measured. The expression of cGAS, STING, and MGF360-9L was analyzed through Western blotting. (C) HEK-293T cells cultured in 12-well plates were transfected with MGF360-9L expression plasmid or vector (1 μg/well). After 24 h, cells were transfected with B-DNA (2 μg/well) and incubated for 12 h. The mRNA expression of IFN-β was detected using RT-qPCR. (D) HEK-293T cells cultured in 48-well plates were transfected with various concentrations of MGF360-9L expression plasmid (0, 0.1, or 0.2 μg/well) along with ISRE-Luc (0.05 μg/well) and pRL-TK (0.005 μg/well) plasmids. After 24 h, the cells were treated with IFN-β (1,000 U/ml) for 8 h, and then a luciferase assay was performed. (E) HEK-293T cells cultured in 12-well plates were transfected with various concentrations of MGF360-9L expression plasmid (0, 0.5, 1, or 2 μg/well). After 24 h, cells were treated with IFN-β (2,000 U/ml) for 8 h. The mRNA expression of *ISG15*, *ISG54*, *ISG56*, and *Mx1* was detected using RT-qPCR. Data are presented as the means ± SDs of three independent experiments.

To screen the ASFV proteins that inhibit IFN-β signaling, human embryonic kidney (HEK)-293T cells were transfected with various ASFV protein expression and STAT1/2 luciferase reporter plasmids. Next, a luciferase assay was performed. The results showed that MGF360-9L strongly inhibits the IFN-β-induced activation of STAT1/2 promoter (Fig. 2A).

To determine whether MGF360-9L inhibits the activation of IFN-β through the cGAS–STING pathway, HEK-293T cells were transfected with cGAS, STING, and different doses of MGF360-9L expression plasmids, along with an IFN-β reporter plasmid. Then, a luciferase assay was performed. The results showed that the IFN-β promoter was activated through the cotransfection of cGAS and STING expression plasmids. However, MGF360-9L overexpression failed to inhibit the cGAS–STING-induced activation of the IFN-β promoter (Fig. 2B). In addition, MGF360-9L did not reduce the B-DNA-induced transcriptional activation of IFN-β (Fig. 2C). Thus, the results revealed that MGF360-9L does not inhibit the cGAS–STING-induced production of IFN-β.

To investigate the involvement of ASFV MGF360-9L in IFN-β signaling, HEK-293T cells were transfected with various doses of ASFV MGF360-9L expression and ISRE-luciferase reporter plasmids. Then, a luciferase assay was performed. ASFV MGF360-9L dose-dependently inhibited IFN-β-induced activation of the ISRE promoter (Fig. 2D). To further assess whether MGF360-9L is an inhibitor of IFN-β signaling, HEK-293T cells were transfected with MGF360-9L expression plasmids and then treated with IFN-β. Afterward, the mRNA levels of *ISG15*, *ISG54*, *ISG56*, *and Mx1* were analyzed using RT-qPCR. MGF360-9L inhibited IFN-β-induced ISG transcription in a dose-dependent manner (Fig. 2E). These results confirmed the suppressive function of MGF360-9L in IFN-β signaling.

### ASFV MGF360-9L disrupts ISGF3-mediated ISRE promoter activation.

**FIG 3:**
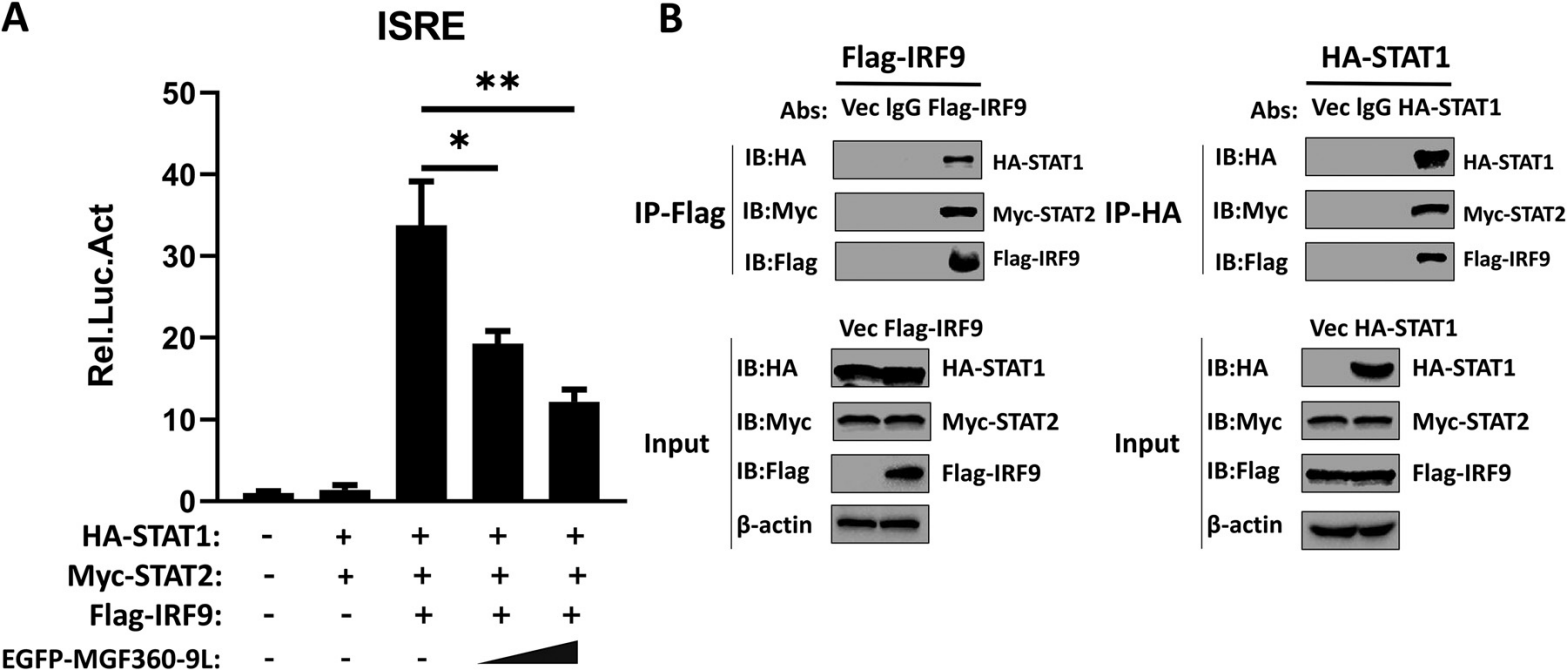
ASFV MGF360-9L inhibits ISGF3-induced ISRE promoter activity. (A) HEK-293T cells cultured in 48-well plates were transfected with various concentrations of EGFP-MGF360-9L expression plasmid (0, 0.1, or 0.2 μg/well) along with HA-*STAT1* (0.2 μg/well), Myc*-STAT2* (0.2 μg/well), Flag*-IRF9* (0.2 μg/well), ISRE-Luc plasmid (0.05 μg/well), and pRL-TK plasmid (0.005 μg/well). After 28 h, a luciferase assay was performed. *: *P < *0.05; **: *P < *0.01. Data are presented as the means ± SDs of three independent experiments. (B) HEK-293T cells were transfected with HA-*STAT1* (5 μg), Myc-*STAT2* (5 μg), and Flag-*IRF9* (5 μg); after 24 h, the cell lysates were subjected to co-IP with anti-Flag or anti-HA antibodies and were immunoblotted with anti-HA, anti-Myc, or anti-Flag antibodies, respectively.

During host defense, STAT1 and STAT2 are phosphorylated in response to IFN-β expression and then combine with IRF9 to form the tripartite transcription factor ISGF3, which enters the nucleus and further enhances the ISRE promoter activity, thereby promoting ISG expression (24). High levels of unphosphorylated STAT1 and STAT2 as well as IRF9 contribute to the formation of unphosphorylated ISGF3, which activates ISRE and significantly increases ISG expression (25, 26). Hence, HEK-293T cells were cotransfected with MGF360-9L expression plasmid (or an empty vector) and STAT1, STAT2, IRF9, and ISRE-Luc plasmids. Then, a luciferase assay was performed. As shown in Fig. 3A, the coexpression of the components of ISGF3 (STAT1, STAT2, and IRF9) significantly activated the ISRE promoter. To confirm whether the expression plasmids of STAT1, STAT2, and IRF9 facilitate the formation of the ISGF3 complex after transfection, HEK-293T cells were transfected with the relevant expression plasmids. The results of coimmunoprecipitation (co-IP) and immunoblotting analyses demonstrated that the cotransfected STAT1, STAT2, and IRF9 indeed contributed toward the formation of the ISGF3 complex in these cells (Fig. 3B). However, ASFV MGF360-9L significantly and dose-dependently inhibited ISGF3-induced activation of the ISRE promoter (Fig. 3A). Thus, ASFV MGF360-9L may target the ISGF3 complex to inhibit IFN-β signaling.

### Construction of ASFV-Δ360-9L and *in vitro* evaluation of its biological characteristics.

**FIG 4:**
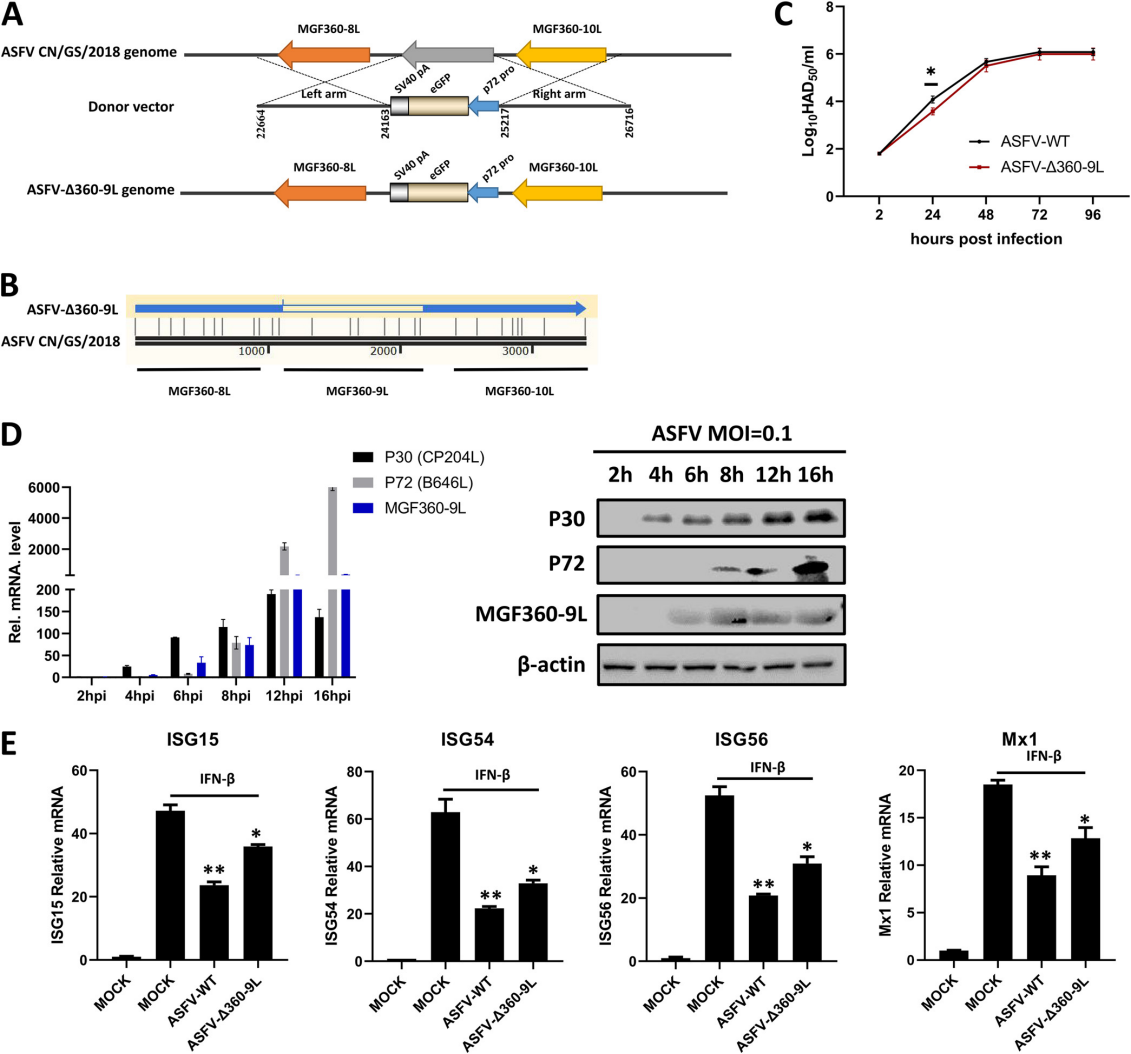
Biological characteristics of ASFV-Δ360-9L and ASFV-WT *in vitro*. (A) The strategy of constructing the MGF360-9L deletion virus by homologous recombination. The specific steps are detailed in Materials and Methods. (B) Results of the partial sequence alignment of ASFV-WT and ASFV-Δ360-9L after whole-genome sequencing (only 22,664–26,716 of ASFV CN/GS/2018). The results of entire genome sequencing are shown in Data Set S1 in the supplemental material. (C) PAMs were infected with ASFV-WT (MOI = 0.01) or ASFV-Δ360-9L (MOI = 0.01), and the viral titer in the sample was determined using the HAD_50_ method at the indicated times after infection. (D) The timing of ASFV MGF360-9L gene expression. PAMs were infected with ASFV (MOI = 0.1), and the cells were collected at specified time points after infection (2, 4, 6, 8, 12, and 16 hpi). The mRNA expression level of *MGF360-9L*, *P30*, and *P72* were detected using qPCR. Porcine GAPDH was used as an internal loading control. The production of MGF360-9L, P30, and P72 proteins was detected by Western blotting. β-actin was used as a loading control. P30 is an early protein encoded by ASFV *CP204L*. P72 is a late protein encoded by ASFV *B646L*. (E) PAMs were infected with ASFV-WT (MOI = 0.1) or ASFV-Δ360-9L (MOI = 0.1) for 8 h and then treated with IFN-β (1,000 U/ml) for 8 h before analyzing the mRNA levels of *ISG15*, *ISG54*, *ISG56*, and *Mx1* using RT-qPCR. Porcine GAPDH was used as an internal loading control. Data are presented as the means ± SDs of three independent experiments.

To study the role of endogenous MGF360-9L in ASFV infection, *MGF360-9L* was deleted from the whole genome of the parent ASFV CN/GS/2018 strain through homologous recombination. Thus, we constructed the recombinant ASFV strain ASFV-Δ360-9L (Fig. 4A). The whole-genome sequencing and analysis of ASFV-Δ360-9L confirmed that *MGF360-9L* was successfully knocked out at an accurate position. Furthermore, no undesirable genome changes occurred (Fig. 4B and Data Set S1 in the supplemental material). The genome sequence was submitted to GenBank and got accession number: OL310288.

10.1128/mBio.02330-21.2 DATA SET S1 Whole-genome sequencing data of ASFV-Δ360-9L. Download Data Set S1, ZIP file, 2.8 MB. Copyright © 2022 Zhang et al. 2022 Zhang et al. https://creativecommons.org/licenses/by/4.0/ This content is distributed under the terms of the Creative Commons Attribution 4.0 International license.

To explore the effect of MGF360-9L on ASFV replication *in vitro*, PAMs were infected with wild-type ASFV (ASFV-WT) or ASFV-Δ360-9L. The viral titer was determined using the 50% hemadsorption dose (HAD_50_) method, and the growth curve was drawn. The results showed that compared with the parent strain, the deletion of *MGF360*-9L reduced the replication level of ASFV-Δ360-9L in PAMs at 24 hpi. The viral titer differences at 24 hpi might have been statistically significant but the difference is unlikely to be biologically relevant. At 48 hpi, the replication of ASFV-Δ360-9L was consistent with that of ASFV-WT (Fig. 4C). In addition, the expression time of ASFV MGF360-9L was later than that of ASFV P30—an early expression protein—but earlier than ASFV P72—a late expression protein (Fig. 4D).

PAMs were infected with ASFV-Δ360-9L and its parental virus (ASFV-WT) and were then treated with IFN-β before measuring the expression levels of ISG using RT-qPCR. ASFV-Δ360-9L had a weaker inhibitory effect on IFN-β-induced transcriptional upregulation of *ISG15*, *ISG54*, *ISG56*, and *Mx1* than ASFV-WT (Fig. 4E). Thus, MGF360-9L may play a role in the inhibition of IFN-β activation of ISGs in ASFV infection.

### MGF360-9L degrades STAT1 and STAT2 through apoptosis and ubiquitin–proteasome pathways, respectively.

**FIG 5:**
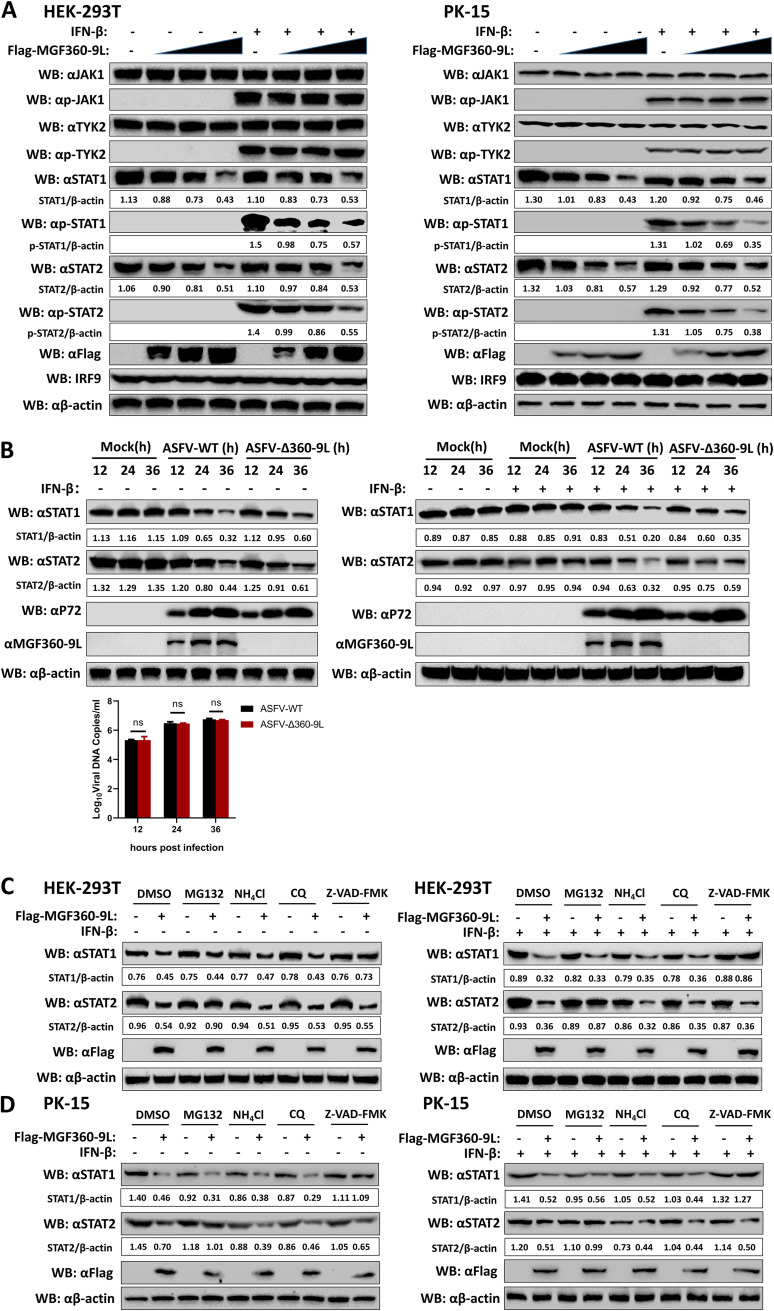
ASFV MGF360-9L protein degrades STAT1 and STAT2 through apoptosis and the ubiquitin–proteasome pathway, respectively. (A) ASFV MGF360-9L downregulates the expression of endogenous STAT1 and STAT2 proteins. HEK-293T and PK-15 cells were transfected with Flag-MGF360-9L (0, 0.5, 1, and 2 μg) and were incubated for 24 h. Then IFN-β treatment was performed (1,000 U/ml) for 4 h. The cell lysates were analyzed through immunoblotting using the indicated antibody. (B) Effects of ASFV-WT and ASFV-Δ360-9L on endogenous STAT1 and STAT2 in PAMs. PAMs were infected with ASFV-WT (MOI = 1) or ASFV-Δ360-9L (MOI = 1) for 12, 24, or 36 h and were then treated with IFN-β (1,000 U/ml) for 4 h. The cell lysates were analyzed via immunoblotting using the indicated antibody. In addition, PAMs were infected with ASFV-WT (MOI = 1) or ASFV-Δ360-9L (MOI = 1) for 12, 24, and 36 h before samples were collected to detect the copy number of ASFV. (C) and (D) Effects of inhibitors on the MGF360-9L-mediated reduction of STAT1 and STAT2. HEK-293T cells (C) or PK-15 cells (D) were transfected with Flag-MGF360-9L expression plasmid (2 μg/well). After 18 h of transfection, the cells were treated with IFN-β (1,000 U/ml) and the indicated inhibitors, namely, MG132 (50 μM), NH_4_Cl (20 mM), CQ (100 μM), or Z-VAD-FMK (50 μM), for 6 h before immunoblotting. DMSO (2 μl/well) was used as the blank control for the inhibitors.

The downregulation of IFN-activated signal transduction molecules is a common defense mechanism used by many viruses (27, 28). HEK-293T and porcine kidney (PK)-15 cells were transfected with various doses of MGF360-9L expression plasmid, and the effect of MGF360-9L on endogenous protein and phosphorylation level of node molecules in JAK/STAT pathway were evaluated via immunoblotting. MGF360-9L dose-dependently reduced the protein levels of endogenous STAT1 and STAT2, with or without IFN-β treatment (Fig. 5A). To investigate the effect of MGF360-9L on STAT1 and STAT2 expression in ASFV infection, PAMs were infected with ASFV-WT or ASFV-Δ360-9L for different lengths of times, and then the cell lysates were analyzed via immunoblotting. ASFV-WT inhibited the expression of STAT1 and STAT2 more strongly than ASFV-Δ360-9L. These results suggested that MGF360-9L reduces STAT1 and STAT2 levels during ASFV infection (Fig. 5B).

HEK-293T and PK-15 cells were transfected with the MGF360-9L expression plasmid and then treated with different protein-degradation pathway inhibitors to explore the mechanism through which MGF360-9L reduces endogenous STAT1 and STAT2 levels. Immunoblotting results revealed that MGF360-9L-mediated degradation of STAT1 was completely inhibited by the apoptotic inhibitor Z-VAD-FMK but not by the proteasome inhibitor MG132 or the lysosomal inhibitors NH_4_Cl and chloroquine (CQ) (Fig. 5C and D). In addition, the proteasome inhibitor MG132 completely inhibited MGF360-9L-mediated STAT2 degradation. However, lysosome inhibitors NH_4_Cl and CQ and the apoptotic inhibitor Z-VAD-FMK failed to inhibit STAT2 degradation (Fig. 5C and 5D). These results suggested that MGF360-9L degrades STAT1 through the apoptotic pathway and STAT2 through the ubiquitin–proteasome pathway.

### ASFV MGF360-9L interacts with STAT1 and STAT2.

**FIG 6:**
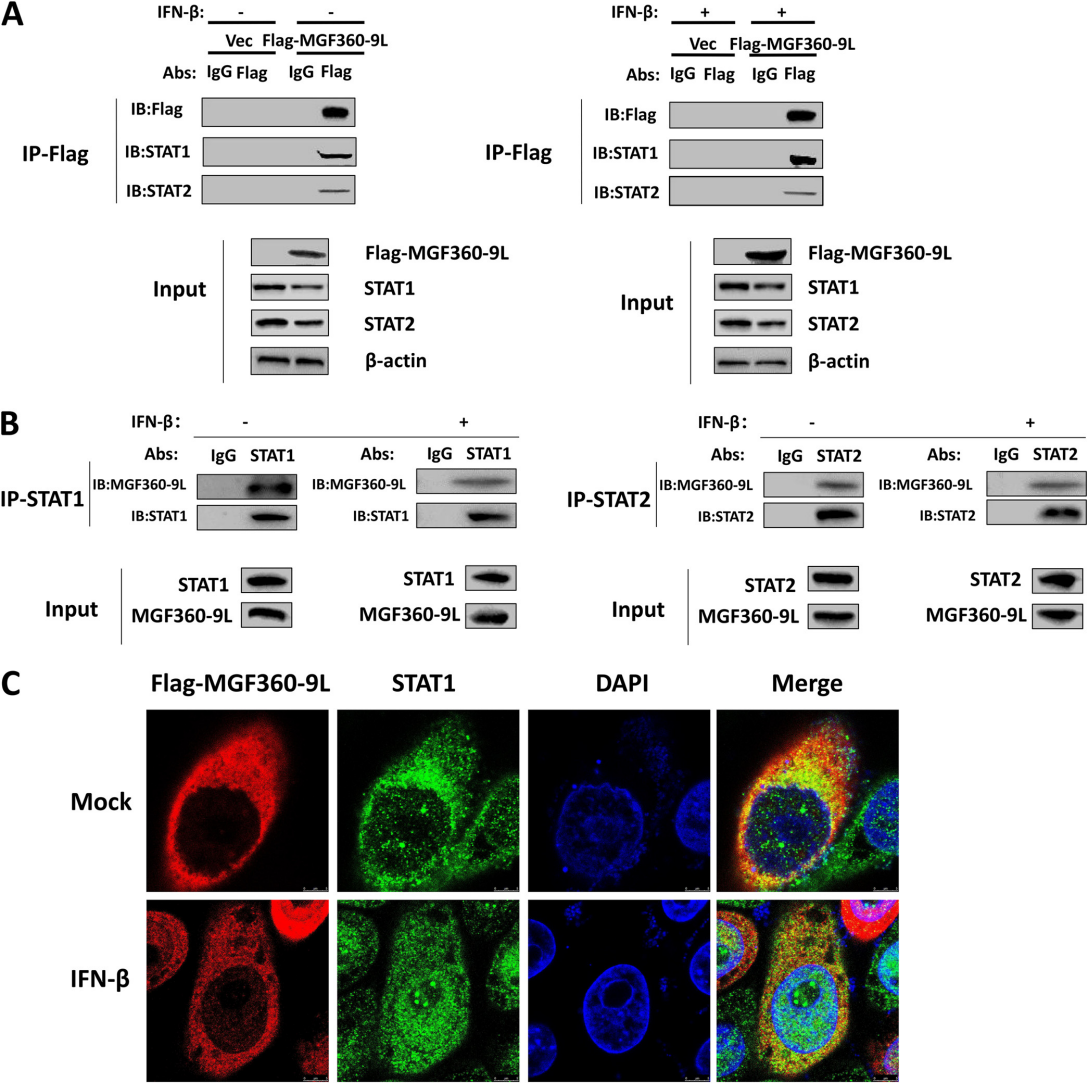
ASFV MGF360-9L interacts with STAT1 and STAT2. (A) MGF360-9L interacts with STAT1 and STAT2. PK-15 cells were transfected with Flag-MGF360-9L (7 μg) expression plasmids. After 24 h, the cells were treated with IFN-β (1,000 U/ml) for 4 h before co-IP with anti-Flag antibodies. The presence of STAT1 and STAT2 proteins were analyzed through immunoblotting using anti-STAT1 and anti-STAT2 antibodies. (B) MGF360-9L protein interacts with STAT1 and STAT2 during ASFV infection. PAMs were infected with ASFV (MOI = 0.5) for 12 h and then left untreated or were treated with IFN-β (1,000 U/ml) for 4 h. Co-IP was performed using the anti-STAT1 or anti-STAT2 antibody. The presence of MGF360-9L proteins was analyzed through immunoblotting using an anti-MGF360-9L antibody. (C) Colocalization of MGF360-9L with STAT1. PK-15 cells were transfected with Flag-MGF360-9L (2 μg) expression plasmid. After 24 h, the cells were treated with IFN-β (1,000 U/ml) for 4 h before observation under a confocal microscope.

Previous studies have shown that several viral proteins inhibit IFN signaling by interacting with the components of ISGF3 (26, 29). PK-15 cells were transfected with MGF360-9L expression plasmid or an empty vector to investigate whether MGF360-9L interacts with endogenous STAT1 and STAT2. The results of co-IP and immunoblotting analyses showed that MGF360-9L interacted with endogenous STAT1 and STAT2 in PK-15 cells, with or without IFN-β treatment (Fig. 6A). To further determine whether MGF360-9L interacts with STAT1 and STAT2 during ASFV infection, ASFV-infected PAMs were immunoprecipitated with anti-STAT1 or anti-STAT2 monoclonal antibodies and were examined for the presence of MGF360-9L using an anti-MGF360-9L polyclonal antibody. The results showed that STAT1 and STAT2 interact with the endogenous MGF360-9L protein in ASFV-infected PAMs, with or without IFN-β treatment (Fig. 6B). PK-15 cells were transfected with the Flag-MGF360-9L expression plasmid, and the localization of MGF360-9L and STAT1 proteins was observed under a confocal microscope. In the absence of IFN-β treatment, MGF360-9L and STAT1 proteins were colocalized in the cytoplasm. After IFN-β treatment, a proportion of STAT1 entered the nucleus from the cytoplasm and the remaining STAT1 proteins in the cytoplasm were still colocalized with MGF360-9L (Fig. 6C). These results confirmed that STAT1 and STAT2 are the interaction partners of the ASFV MGF360-9L protein.

### Assessment of ASFV-Δ360-9L virulence in pigs.

**FIG 7:**
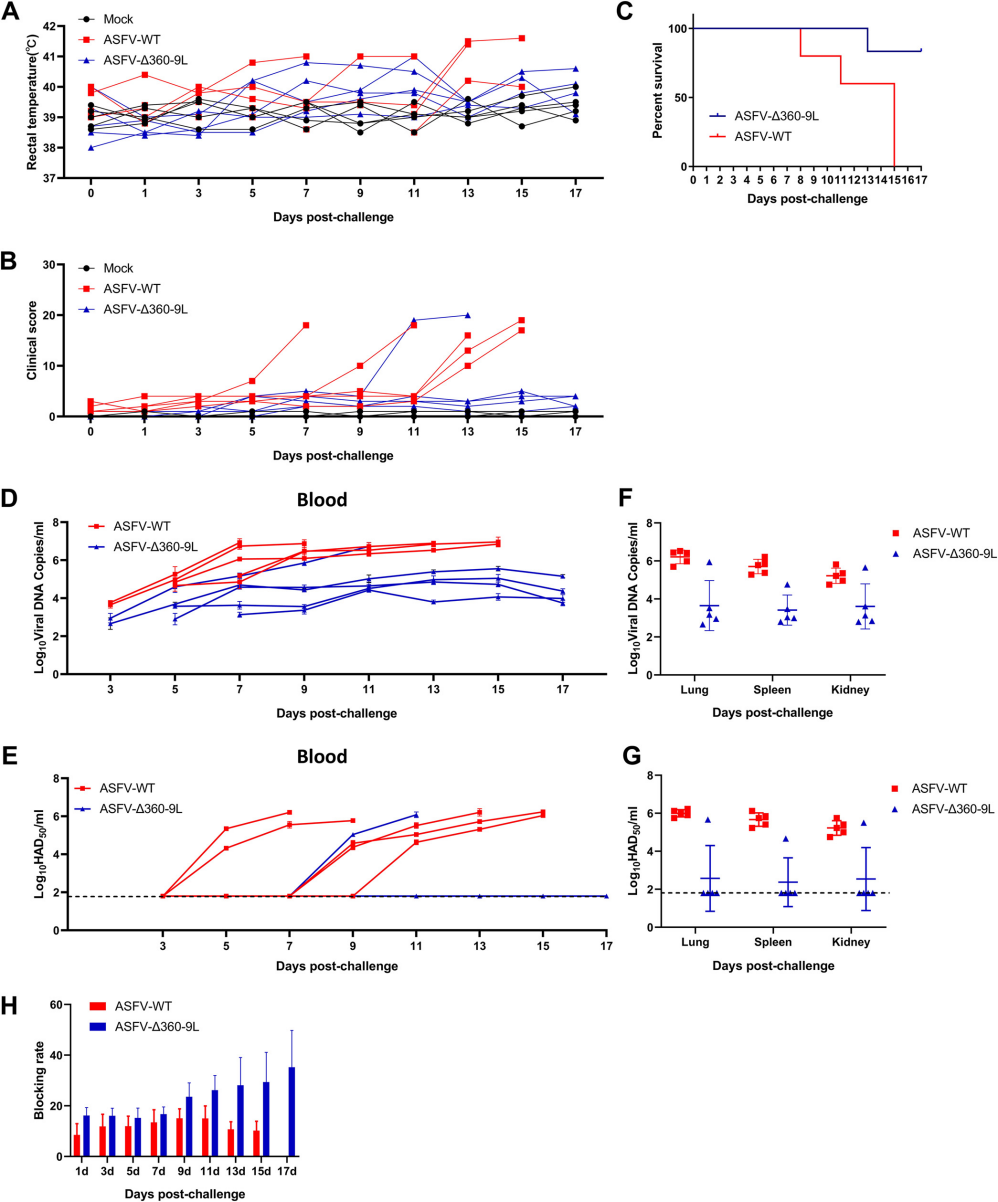
Deletion of MGF360-9L reduces ASFV virulence in pigs. (A) The kinetics of body temperature values in pigs intramuscularly injected with 1 HAD_50_ of ASFV-WT (shown in red), ASFV-Δ360-9L (shown in blue), or mock-inoculation solution (shown in black). (B) The clinical scores of pigs injected with 1 HAD_50_ of ASFV-WT, ASFV-Δ360-9L, or mock-inoculation solution. (C) The survival rate of pigs intramuscularly injected with 1 HAD_50_ of ASFV-WT or ASFV-Δ360-9L. (D) qPCR-detected viral load in the blood of pigs infected with 1 HAD_50_ of ASFV-WT or ASFV-Δ360-9L. (E) HAD_50_-method-detected viral titer in the blood of pigs infected with 1 HAD_50_ of ASFV-WT or ASFV-Δ360-9L detected using the HAD_50_ method. (F) qPCR-detected viral load in different tissues of pigs intramuscularly infected with 1 HAD_50_ of ASFV-WT or ASFV-Δ360-9L. (G) HAD50-method-detected viral titer in different tissues of pigs intramuscularly injected with 1 HAD_50_ of ASFV-WT or ASFV-Δ360-9L detected using the HAD_50_ method. (H) ELSIA-detected anti-p30 antibody levels in pigs intramuscularly injected with 1 HAD_50_ of ASFV-WT or ASFV-Δ360-9L. Each curve or icon (circle, square, and triangle) represents the value of each animal in each group. The sensitivity of virus detection was ≥log_10_ 1.8 HAD_50_/ml. Data are presented as the means ± SDs of three independent experiments.

Pigs (*n* = 5) were intramuscularly injected with 1 HAD_50_ of the parent virus ASFV CN/GS/2018 (ASFV-WT) or the recombinant virus ASFV-Δ360-9L to evaluate the virulence of ASFV-Δ360-9L. Clinical signs were recorded daily from day 0 (the day of injection). All five pigs infected with ASFV CN/GS/2018 showed increased body temperature, similar to that near death, and clinical symptoms associated with ASF such as anorexia, stumbling gait, and diarrhea (Fig. 7A and B). One of the five pigs infected with ASFV-Δ360-9L showed clinical symptoms associated with ASF and died at 13 days postinfection (dpi). One of the other four pigs developed a low fever at 11 dpi but survived until the end of the experiment. The remaining three pigs had normal body temperature or developed fever for only a short period, and then the temperature returned to normal. All mock-inoculated pigs showed no specific clinical symptoms (Fig. 7A and 7B). The clinical symptoms of experimental animals were comprehensively evaluated according to the method described by King et al. (30). The clinical scores of surviving pigs infected with ASFV-Δ360-9L were lower than that of the pigs infected with ASFV-WT (Fig. 7B). At the end of the experiment, all pigs inoculated with ASFV-WT died or were on the verge of death at 8–15 dpi (survival rate: 0%). On the other hand, one pig infected with ASFV-Δ360-9L died and four survived (survival rate: 80%; Fig. 7C).

Four of the five pigs infected with ASFV-Δ360-9L had a remarkably lower viral load in blood than the pigs infected with ASFV-WT (Fig. 7D). However, using the HAD_50_ method, high viral titers were detected only in the blood of those pigs that died after being injected with ASFV-WT or ASFV-Δ360-9L; the live virus was not detected in the blood of pigs that survived after ASFV-Δ360-9L infection (Fig. 7E). Tissue samples from the lungs, spleen, and kidneys of pigs infected with ASFV-WT showed a high viral load. Of the five pigs infected with ASFV-Δ360-9L, the one pig that died during the experiment showed a high viral load (Fig. 7F). The remaining four pigs had a relatively lower viral load in the lung, spleen, and kidney samples than pigs infected with ASFV-WT (Fig. 7F). Similar to the findings regarding the viral titers in blood, high viral titers were noted in the lung, spleen, and kidney samples of the pigs that died after infection with ASFV-WT or ASFV-Δ360-9L. In contrast, no live virus was detected in pigs that survived ASFV-Δ360-9L infection (Fig. 7G). Furthermore, we explored the specific antibody response induced by ASFV-Δ360-9L. The P30 antibody level of pigs infected with ASFV-Δ360-9L appear to be increasing from 9 dpi (Fig. 7H). These results indicated that the deletion of *MGF360-9L* weakens the virulence of the ASFV CN/GS/2018 strain in pigs.

### ASFV-Δ360-9L infection causes mild pathological injury in pigs compared with ASFV-WT.

**FIG 8:**
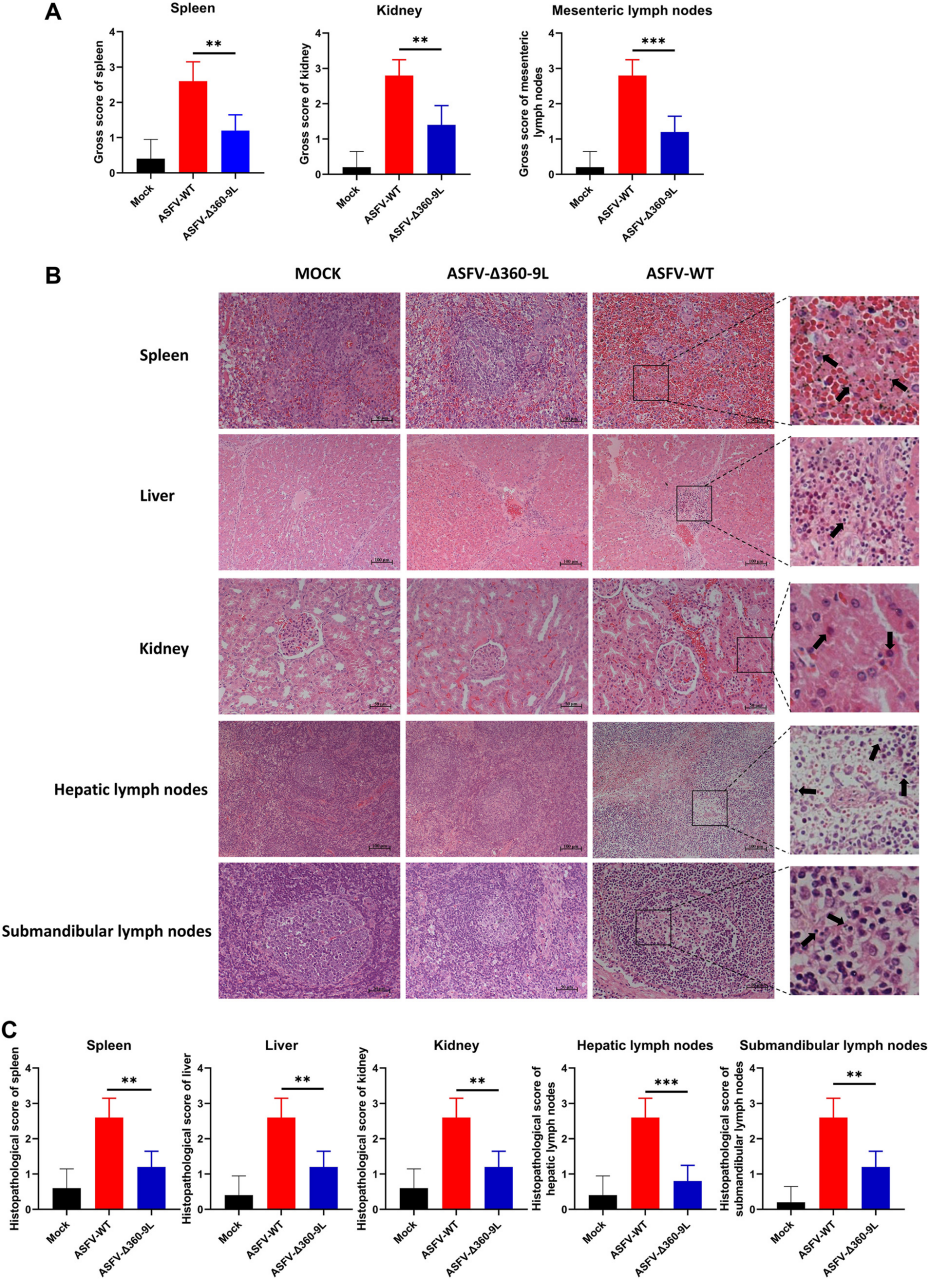
Deletion of *MGF360-9L* reduces the pathogenicity of ASFV. (A) Gross scores of the spleen, kidney, and Mesenteric lymph node samples of pigs infected with ASFV-WT (1 HAD_50_) or ASFV-Δ360-9L (1 HAD_50_). (B) Histopathological changes noted in the spleens, liver, kidneys, hepatic lymph nodes, and submandibular lymph nodes of pigs infected with ASFV-WT (1 HAD_50_) or ASFV-Δ360-9L (1 HAD_50_). (C) Histopathological scores of the spleens, liver, kidneys, hepatic lymph nodes, and submandibular lymph nodes of pigs infected with ASFV-WT (1 HAD_50_) or ASFV-Δ360-9L (1 HAD_50_). The evaluation criteria of pathological injury were based on the study of Galindo-Cardiel in 2013. The black arrows represent cell necrosis, nuclear pyknosis, and fragmentation. Data are presented as the means ± SDs of three independent experiments.

The spleens of pigs infected with ASFV-WT showed swelling and congestion; the kidneys were darkened, which might have been congested. Moreover, the mesenteric lymph nodes showed remarkable congestion. Conversely, the spleens, kidneys, and mesenteric lymph nodes of pigs that survived ASFV-Δ360-9L infection showed mild pathological changes (Fig. S1 in the supplemental material). The spleens, kidneys, and mesenteric lymph nodes of all pigs infected with ASFV-WT or ASFV-Δ360-9L or those of the pigs that received mock injection were scored grossly. The gross scores were significantly lower for pigs infected with ASFV-Δ360-9L than for those infected with ASFV-WT (Fig. 8A).

10.1128/mBio.02330-21.1 FIG S1 Gross pathological changes in the spleens, kidneys, and mesenteric lymph nodes of pigs infected with ASFV-WT (1 HAD50) or ASFV-Δ360-9L (1 HAD50). The red arrows indicate the focal lesion sites. Download FIG S1, TIF file, 2.7 MB. Copyright © 2022 Zhang et al. 2022 Zhang et al. https://creativecommons.org/licenses/by/4.0/ This content is distributed under the terms of the Creative Commons Attribution 4.0 International license.

Fig. 8B and C present the histopathological damage and scores obtained from the significant organs of the pigs. In the spleen sections of pigs infected with ASFV-WT, red and white pulp structures were unclear; extensive bleeding was accompanied by lymphocyte necrosis and nuclear fragmentation. In the liver sections of pigs infected with ASFV-WT, congestion was observed in the blood vessels and sinusoid spaces; many inflammatory cells infiltrated in the portal area. In the renal sections of pigs infected with ASFV-WT, hemorrhage in the renal interstitium and necrosis of tubular epithelial cells were noted. In the hepatic and submandibular lymph node sections of pigs infected with ASFV-WT, the structure of lymph nodules was blurred; lymphocytes were necrotic and absent. In the sections of the spleens, liver, kidneys, hepatic lymph nodes, and submandibular lymph nodes of pigs surviving after ASFV-Δ360-9L infection, these injuries were milder or disappeared. The histopathological scores of the spleens, liver, kidneys, hepatic lymph nodes, and submandibular lymph nodes of pigs infected with ASFV-Δ360-9L were significantly lower than those of pigs infected with ASFV-WT (Fig. 8C). The results confirmed that the degree of pathological damage in the spleens, liver, kidneys, hepatic lymph nodes, and submandibular lymph nodes of pigs infected with ASFV-Δ360-9L was less than that of pigs infected with ASFV-WT.

## DISCUSSION

ASFV MGF360 and MGF530/505 have been reported to inhibit the expression of type I IFN and suppress the antiviral effect of IFN and increase the proliferation efficiency of the virus in host cells by prolonging the survival duration of the infected cells (6, 16, 19, 20). As the members of the MGF360 family, A276R and MGF360-12L inhibit type I IFN expression (31, 32). Our study confirmed that MGF360-9L is a negative regulator of IFN-β-induced antiviral genes and that MGF360-9L is a virulence factor of ASFV (Fig. 2, 4 and 7, and 8).

**FIG 9:**
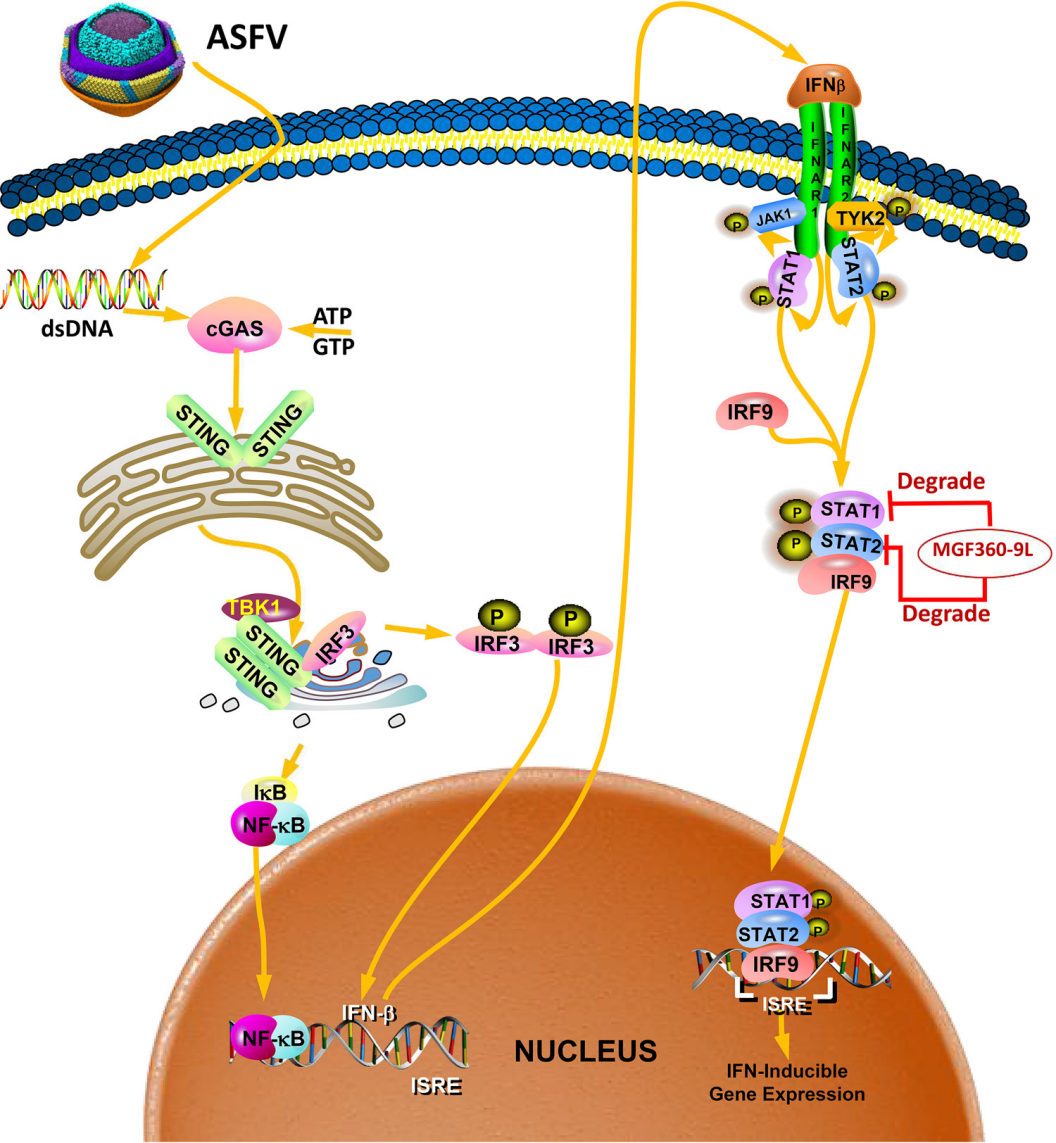
Schematic diagram of the mechanism of MGF360-9L-mediated IFN-β signaling inhibition. ASFV MGF360-9L interacts with STAT1/2 and degrades STAT1/2 to help the virus escape the host immune response.

The ability of a virus to regulate the JAK/STAT pathway is essential for the occurrence and maintenance of infection (9). Reports have indicated that viruses target the components of the transcription factor complex ISGF3 (STAT1, STAT2, and IRF9) to inhibit type I IFN signaling (26, 27). For instance, the porcine epidemic diarrhea virus disrupts type I IFN response by inducing STAT1 degradation (27). Furthermore, the nonstructural NS5 protein of Zika virus binds to and targets human STAT2 to inhibit type I IFN signaling (28). The interaction between nonstructural protein 11 from porcine reproductive and respiratory syndrome virus (PRRSV) and IRF9 block IFN signaling by impairing the formation and nuclear translocation of ISGF3 (26). In addition, ASFV induces STAT1 and STAT2 degradation to counteract Type I IFNs signaling (13). Our data showed that ASFV MGF360-9L interacts with, and subsequently degrades, STAT1 and STAT2, thereby inhibiting IFN-β signaling (Fig. 5 and 6). Protein degradation is one of the primary strategies to regulate protein function in biological processes. The ubiquitin–proteasome system, autophagy-lysosomal pathway, and apoptosis are three main protein-degradation strategies. The PRRSV E protein degrades porcine cholesterol 25-hydroxylase, via the ubiquitin–proteasome pathway (33). ASFV-MGF505-7R degrades STING through the autophagy-lysosomal pathway (34). The results of this study indicated that MGF360-9L degraded STAT1 through the apoptotic pathway and STAT2 through the ubiquitin–proteasome pathway (Fig. 5C). However, which host proteins are involved in the MGF360-9L-mediated degradation of STAT1 and STAT2 remain unknown. Compared with ASFV-WT, the ability of ASFV-Δ360-9L to degrade STAT1 and STAT2 was significantly reduced, but it did not disappear (Fig. 5). In addition, the inhibitory effect of ASFV-Δ360-9L on IFN-β-induced ISG transcriptional upregulation was weaker than that of ASFV-WT, but this also did not disappear (Fig. 4). This indicates that in addition to MGF360-9L protein, there might be other viral proteins that can inhibit IFN-β signaling during ASFV infection. Our results uncovered a new mechanism through which ASFV inhibits the host innate immune signaling pathway (Fig. 9).

Gene deletion is an effective way to develop attenuated vaccines against ASFV. The deletion of a single or multiple gene/s has been demonstrated to exert a protective effect during ASFV infection (35–37). The deletion of *DP148R*, *I177L*, or *9GL* (potentiated by the additional deletion of *UK*) attenuates the virulence of highly virulent ASFV strains and protects against virulent parent viruses (35–38). Furthermore, the deletion or interruption of some *MGF360* and *MGF530/505*, including *MGF360-9L*, from a virulent ASFV isolate reduces virulence during infection in domestic pigs and induces a protective response (15, 19). The present study confirmed that the deletion of *MGF360-9L* from the ASFV CN/GS/2018 (ASFV-Δ360-9L) partially attenuates the virulence of this virus in pigs (Fig. 7 and 8).

Notably, the viral titer differences between ASFV-WT and ASFV-Δ360-9L at 24 hpi in PAMs might have been statistically significant but the difference is unlikely to be biologically relevant (Fig. 4C). The difference may be related to the inhibition of innate immunity by MGF360-9L. In addition, the viral copies in blood and tissues (lung, spleen and kidney) in the survivors among the pigs infected with ASFV-Δ360-9L was significantly lower than that in pigs infected with ASFV-WT (Fig. 7D and F). High viral titers were detected by HAD_50_ assay in the blood and tissues of pigs infected with ASFV-WT; however, live viruses could not be detected in pigs that survived ASFV-Δ360-9L infection (Fig. 7E and 7G). Interestingly, most of the pigs infected with ASFV-WT had symptoms of high fever before death, but this was not observed in pigs that survived ASFV-Δ360-9L infection. A previous study has shown that pigs infected with ASFV-G-DI177L survived and had normal body temperature (36). Therefore, elevated body temperature may be a sign of the disease in pigs infected with ASFV.

In conclusion, MGF360-9L interacts with STAT1 and STAT2 to inhibit IFN-β signaling via the degradation of STAT1 through apoptosis and STAT2 through the proteasome pathway. In addition, the deletion of *MGF360-9L* attenuates ASFV. This study reveals a new mechanism used by ASFV to evade the host immunity and guides the development of safer and more reliable attenuated recombinant ASFV vaccines.

## MATERIALS AND METHODS

### Animal experiments and ethics statement.

Animal experiments were performed at Lanzhou Veterinary Research Institute of the Chinese Academy of Agricultural Sciences (Lanzhou, China). Landrace pigs of aged approximately 75 days and weighing 30–35 kg were obtained from a high-health farm. All pigs examined were negative for PRRSV, pseudorabies virus, or porcine epidemic diarrhea virus. In this experiment, pigs were injected with ASFV-Δ360-9L (1 HAD_50_) or ASFV CN/GS/2018 (1 HAD_50_). After injection (0 dpi), rectal temperature and clinical signs were monitored daily and scored as described (30) previously. Blood samples were collected from all pigs before 0 dpi, after injection, and at different time points after injection.

All animal experiments (including the pig euthanasia procedure) were performed in accordance with the regulations and guidelines of Lanzhou Veterinary Research Institute (Chinese Academy of Agriculture Science) institutional animal care and the Assessment and Accreditation of Laboratory Animal Care International and the Institutional Animal Care and Use Committee guidelines (License No. SYXK [GAN] 2014–003).

All experiments involving live ASFVs were performed in the biosafety level 3 facilities of Lanzhou Veterinary Research Institute, Chinese Academy of Agricultural Sciences.

### Cell culture, virus, antibodies, and reagents.

PK-15 cells were cultured in minimum Eagle’s medium containing 10% fetal bovine serum (FBS, Sigma-Aldrich, St. Louis, MO, USA). HEK-293T cells were cultured in Dulbecco’s modified Eagle’s medium (Life Technologies, Grand Island, NY, USA) containing 10% FBS. PAMs were prepared using bronchoalveolar lavage as described previously (39) and cultured in Roswell Park Memorial Institute 1640 medium containing 10% porcine serum. Porcine bone marrow-derived macrophage (BMDM) cells were prepared as described previously (40) and cultured in Roswell Park Memorial Institute 1640 medium containing 10% porcine serum. Cells were grown in a humidified atmosphere containing 5% CO_2_ at 37°C.

ASFV CN/GS/2018 was provided by the Lanzhou Veterinary Research Institute, Chinese Academy of Agricultural Sciences. ASFV CN/GS/2018 is denoted by ASFV or ASFV-WT in the text. Titration of the virus was performed using the hemadsorption assay; the results are presented as the number of HAD_50_ per milliliter.

Rabbit anti-MGF360-9L and anti-P72 (B646L) polyclonal antibody, Mouse anti-P30 (CP204L) and P30-HRP(CP204L) monoclonal antibody were prepared and provided by our laboratory. The following mouse monoclonal antibodies were used: anti-Myc (2276S), β-Actin (3700S), anti-FLAG (8146S), and anti-JAK1 (50996S); the following rabbit monoclonal antibodies were used: anti-FLAG (14793S), anti-HA (3724S), anti-STAT1 (14994S), anti-p-STAT1 (19167S), anti-STAT2 (72604S), anti-p-STAT2 (88410S), anti-IRF9 (76684S), anti-TYK2 (14193S), anti-p-JAK1 (74129S), and anti-p-TYK2 (68790S). All antibodies were purchased from Cell Signaling Technology (Danvers, MA, USA). Horseradish peroxidase (HRP)-conjugated goat antirabbit IgG (H+L; SA00001-2), HRP-conjugated goat antimouse IgG (H+L; SA00001-1), mouse monoclonal antibody against GFP (66002-1-Ig) were purchased from Proteintech Group Co., Ltd. (Chicago, IL, USA). Protein G Sepharose (17061801) were purchased from GE Healthcare Bio-sciences AB (Pittsburgh, PA, USA). HRP-conjugated goat antimouse IgG (SE131) was purchased from Beijing Solarbio Science And Technology Co., Ltd (Beijing, China). MG132, chloroquine, and Z-VAD-FMK were purchased from MedChemExpress (Monmouth Junction, NJ, USA). Lipofectamine2000 transfection reagent (11668019) was purchased from Thermo Fisher Scientific. jetPRIME and jetPEI-Macrophage were purchased from Polyplus-Transfection SA (Strasbourg, France).

### ASFV-Δ360-9L construction.

The recombinant ASFV-Δ360-9L were generated by homologous recombination method using the parental ASFV genome and a recombination transfer vector, as described previously (41). First, the p72 promoter sequence was amplified through PCR. *GFP* was amplified sing the peGFP-N1 vector and ligated through fusion PCR to construct the enhanced GFP (EGFP)-screening expression cassette. Then, 1.5-kb upstream and downstream sequences of *MGF360-9L* were designed as homologous recombination arms and cloned into the skeleton vector pUC57. The nucleotide sequence of the left recombinant arm was the same as that of ASFV CN/GS/2018 nucleotides 22,664–24,163, and the right recombinant arm was the same as that of ASFV CN/GS/2018 nucleotides 25,217–26,716. Next, P72-eGFP-SV40 polyA was inserted into the recombinant transfer vector’s left and right arm gene sequences to construct the homologous recombination transfer vector. Consequently, BMDM cells were transfected with the homologous recombinant transfer plasmid pUC-LRΔMGF360-9L-eGFP, and the cells were then infected with ASFV CN/GS/2018. After 24 h, recombinant virus infection was observed under a fluorescence microscope. Finally, the purified ASFV MGF360-9L gene-knockout virus was obtained through limited dilution, expanded culture, purity test, and PCR determination of the target gene (Primer 1: ATGGTCCTCTCTCTGCAGA; Primer 2: GATAACATGCTGGCAATGAACGAG). The deleted *MGF360-9L* was located at 24,164–25,216 in the whole gene sequence of ASFV CN/GS/2018.

### ASFV complete genome sequencing and analysis.

PAMs were seeded as described and were infected with ASFV-WT or ASFV-Δ360-9L. DNA was isolated as described above from cells infected with the either of the viruses. Then, whole genome sequencing of the ASFV was performed (42). The isolated DNA was broken into segments using the Covaris ultrasonic crusher. In addition, a DNA library was prepared via terminal repair, adding poly-A tail, adding sequencing connector, purification, and PCR amplification. After the completion of library construction, the library was initially quantified using Qubit v.2.0 and diluted to 2 ng/μl; then, the insert fragment size of the library was detected via Agilent 2100. The effective concentration of the library was accurately quantified using the qPCR to ensure library quality. The DNA library was sequenced using Illumina HiSeq. The sequencing data were assembled using the SPAdes (v.3.13.0) software. The complete and accurate deletion *MGF360-9L* in ASFV-Δ360-9L was confirmed using whole-genome sequencing. There were no undesirable genetic changes in the virus (Data Set S1 in the supplemental material).

### Plasmid construction.

The luciferase reporter plasmid ISRE-Luc has been described previously (43). pRL-TK plasmid (Promega) was used as an internal control to normalize the transfection efficiency. MGF360-9L open reading frame was amplified from the parent ASFV CN/GS/2018 strain genome and was cloned into the pCDNA3.1 vector with Flag or EGFP tags. Plasmids encoding porcine STAT1 (GenBank No: NM_213769.1) and STAT2 (GenBank No: HM462244.1) were constructed by cloning the synthesized sequence into pCDNA3.1 with Myc or HA tags fused to the 3′ end. All constructed plasmids were confirmed through sequencing.

### Transfection and dual-luciferase reporter assays.

To determine the binding activity of promoter, firefly luciferase reporter plasmid (IFN-β-LUC, ISRE-LUC, or STAT1/2-LUC), *Renilla* luciferase reporter pRL-TK, and other expression plasmids were cotransfected into HEK-293T in a 24-well plate. After 24 h of transfection, the cells were treated with IFN-β (1,000 U/ml) or mock-treated for 4 h. Luciferase analysis was performed using a dual-luciferase reporter analysis system (Promega GLOMAX); firefly luciferase activities were normalized and analyzed based on *Renilla* luciferase activity. Each experiment was performed in triplicate.

### Real-time qPCR for determination of relative gene expression.

Total RNA was extracted from PAMs or HEK-293T cells using the TRIzol reagent and was reverse transcribed using the PrimeScript RT kit (TaKaRa). qPCR was performed using the PowerUp SYBR green Master Mix on the ABI StepOnePlus system. All data were analyzed using the StepOnePlus software, and the relative mRNA level of these genes was normalized based on the porcine glyceraldehyde 3-phosphate dehydrogenase (GAPDH) or human GAPDH mRNA level. Furthermore, the relative expression of mRNA was determined based on the comparative cycle threshold (2^−ΔΔCT^) method (44). Table 1 shows the relevant primer sequences.

**TABLE 1:** Primers and oligonucleotides used in this study

Primers	Sequences (5′–3′)
Porcine ISG15-F	GGTGCAAAGCTTCAGAGACC
Porcine ISG15-R	GTCAGCCAGACCTCATAGGC
Porcine ISG54-F	CTGGCAAAGAGCCCTAAGGA
Porcine ISG54-R	CTCAGAGGGTCAATGGAATTCC
Porcine ISG56-F	TCAGAGGTGAGAAGGCTGGT
Porcine ISG56-R	GCTTCCTGCAAGTGTCCTTC
Porcine Mx1-F	AGCGCAGTGACACCAGCGAC
Porcine Mx1-R	GCCCGGTTCAGCCTGGGAAC
Human IFN-β-F	TCTTTCCATGAGCTACAACTTGCT
Human IFN-β-R	GCAGTATTCAAGCCTCCCATTC
Human ISG15-F	CAACTGGCATGGGACCAATG
Human ISG15-R	ATTCCAATGAGCTGGCATCAAG
Human ISG54-F	ACGGTATGCTTGGAACGATTG
Human ISG54-R	AACCCAGAGTGTGGCTGATG
Human ISG56-F	CCTCCTTGGGTTCGTCTACA
Human ISG56-R	GGCTGATATCTGGGTGCCTA
Human Mx1-F	CAGGACATTTGAGACAATCGTG
Human Mx1-R	TCGAAACATCTGTGAAAGCAAG
ASFV P72-F	TGCGATGATGATTACCTT
ASFV P72-R	ATTCTCTTGCTCTGGATAC
ASFV P30-F	CTCCGATGAGGGCTCTTGCT
ASFV P30-R	AGACGGAATCCTCAGCATCTTC
Porcine GAPDH-F1	ACATGGCCTCCAAGGAGTAAGA
Porcine GAPDH-R1	GATCGAGTTGGGGCTGTGACT
Human GAPDH-F1	GAGTCAACGGATTTGGTCGT
Human GAPDH-R1	GACAAGCTTCCCGTTCTCAG

### Real-time qPCR for the detection of ASFV genome copies.

The copy number of ASFV DNA was evaluated through relative quantification as previously described (45). Briefly, ASFV genomic DNA was extracted using QIAamp DNA Mini Kits (Qiagen, Germany) from homogenates or blood, and then qPCR was performed using Pro *Taq* HS Premix Probe qPCR kit (Accurate Biology, China) and a QuantStudio5 system (Applied Biosystems, USA). The target for amplification of the ASFV genome was the conserved p72 gene segment, using the following primers:

ASFV-P72-R: 5′-CTGCTCATGGTATCAATCTTATCGA-3′

ASFV-P72-F: 5′- GATACCACAAGATCAGCCGT-3′

TaqMan: 5′-CCACGGGAGGAATACCAACCCAGTG-3′

Amplification conditions used were as follows: preheating at 95°C for 30 s; 40 cycles at 95°C for 5 s; and annealing at 58°C for 30 s; elongation (72°C). The quantity of the ASFV genome was calculated using a standard curve and expressed as genome copies per milliliter.

### Viral titration.

The samples containing ASFV-WT or ASFV-Δ360-9L were quantified using the HAD_50_ assay as described previously (46), with minor modifications. PAMs were spread onto a 96-well plate. The sample was diluted to 10^−1^, 10^−2,^ 10^−3^, 10^−4^, 10^−5^, 10^−6^, 10^−7^, and 10^−8^ and was added to a 96-well plate. The adsorption of erythrocytes was observed for 7 days. HAD_50_ was calculated according to the Reed–Muench method (47).

### Co-IP.

PAM or PK-15 cells were collected, and the cells were lysed using ethylenediamine tetra-acetic acid (EDTA)-free EASY buffer for 1 h at 4°C; ultrasound was used for 1.5 min with an interval of 5 s. The cell lysate was then incubated with the indicated antibody or control IgG at 4°C overnight. Subsequently, samples were incubated with protein G agarose beads (Roche) for 3 h, washed thrice with EDTA-free EASY buffer (5 min/wash), and boiled in sodium dodecyl sulfate (SDS) loading buffer.

### Immunoblotting.

For Western blotting, the protein was separated using 10% SDS-polyacrylamide gel electrophoresis (75 V, 40 min; 120 V, 60 min) and was then transferred to a nitrocellulose membrane (Pall; 100 V 120 min). Then, the membrane was blocked with 5% skim milk, washed with TBS containing 0.1% Tween 20 (TBST), and incubated with the designated antibodies at 4°C overnight. Next, the membrane was washed with TBST five times (each for 5 min) and incubated with the HRP-conjugated goat antirabbit IgG (Proteintech, SA00001-2) or HRP-conjugated goat antimouse IgG (Proteintech, SA00001-1). Finally, an electrochemiluminescence solution was added to the incubator, and images taken using the Odyssey infrared imaging system.

### Indirect immunofluorescence assay.

Flag-MGF360-9L expression plasmids were transfected into PK-15 cells with the jetPRIME transfection reagent (Polyplus). After 24 h of transfection, the cells were treated with (+) IFN-β (1,000 U/ml) or left untreated (−) for 4 h. The cells were then fixed with 4% paraformaldehyde for 30 min, permeabilized with 0.2% TritonX-100 for 10 min, and blocked with 5% BSA for 1 h. Next, these were incubated with anti-Flag mouse MAb (CST, 8146S) and anti-STAT1 rabbit MAb (CST, 14994S) for 8 h. Then, these were incubated with Alexa Fluor 488 antirabbit and Alexa Fluor 568 antimouse lgG H&L (Abcam) for 2 h and then stained with 4-methyl-6-phenylindole for 10 min. The samples were detected using the Leica SP2 confocal system (Leica Microsystems).

### Histopathological analysis.

The fixed tissues were embedded in paraffin and cut into serial sections (5-μm thick). After dewaxing with xylene and dehydration with ethanol, the samples were stained with hematoxylin and eosin and examined microscopically. Three representative visual fields on each slice were independently evaluated by two pathologists who were blinded to the grouping. The scoring standard of the organ and tissue section were in accordance with the study by Galindo-Cardiel (48).

### Antibody detection.

Microtiter plates were coated (0.1 μg/well) with p30 protein in 0.05 mol/liter carbonate buffer solution (pH 9.6) and incubated overnight at 4°C. The plates were washed thrice with phosphate buffer saline containing 0.1% Tween 20 (PBST), blocked with 200 μl of 1% nonfat milk, and incubated at 4°C for 12 h. After three washes with PBST, 50 μl of the test sample was added into wells containing 50 μl of dilution buffer; then, the plates were incubated for 30 min at 37°C followed by three washes. Next, 100 μl/well of P30-MAb-HRP (1:25,000) was added to each well and incubated at 37°C for 30 min. After the final three washes with PBST, 100 μl/well of 3,3′,5,5′-tetramethylbenzidine substrate was added to each well, and the plates were incubated in the dark for 15 min at 37°C. Finally, 50 μl/well H_2_SO_4_ (2 mol/liter) was used to stop the colorimetric reaction; the OD_450nm_ values were read using an automated ELISA plate reader.

### Statistical analysis.

All *in vitro* experiments were performed at least thrice. Data are presented as the means ± standard deviations (SDs). The statistical significance between groups was determined using the *t* test with GraphPad Prism v.8 (San Diego, CA, USA). *, *P < *0.05, **, *P < *0.01 and ***, *P < *0.001 was considered statistically significant. ns: no significant difference.
